# Deep learning in bladder cancer imaging: A review

**DOI:** 10.3389/fonc.2022.930917

**Published:** 2022-10-20

**Authors:** Mingyang Li, Zekun Jiang, Wei Shen, Haitao Liu

**Affiliations:** ^1^ Department of Urology, Shanghai General Hospital, Shanghai Jiao Tong University School of Medicine, Shanghai, China; ^2^ Ministry of Education (MoE) Key Lab of Artificial Intelligence, Artificial Intelligence (AI) Institute, Shanghai Jiao Tong University, Shanghai, China

**Keywords:** bladder cancer, deep learning, artificial intelligence, medical imaging, computed tomography, magnetic resonance imaging

## Abstract

Deep learning (DL) is a rapidly developing field in machine learning (ML). The concept of deep learning originates from research on artificial neural networks and is an upgrade of traditional neural networks. It has achieved great success in various domains and has shown potential in solving medical problems, particularly when using medical images. Bladder cancer (BCa) is the tenth most common cancer in the world. Imaging, as a safe, noninvasive, and relatively inexpensive technique, is a powerful tool to aid in the diagnosis and treatment of bladder cancer. In this review, we provide an overview of the latest progress in the application of deep learning to the imaging assessment of bladder cancer. First, we review the current deep learning approaches used for bladder segmentation. We then provide examples of how deep learning helps in the diagnosis, staging, and treatment management of bladder cancer using medical images. Finally, we summarize the current limitations of deep learning and provide suggestions for future improvements.

## Introduction

According to the latest statistics from Global Cancer, bladder cancer (BCa) is the tenth most common cancer in the world, with approximately 573,000 new cases and 213,000 deaths in 2020 (1). Early diagnosis and treatment are key to reducing morbidity and mortality associated with BCa (2, 3). In current clinical practice, pathological examination following transurethral resection of bladder tumor (TURBT) and cystoscopy are the gold standard for diagnosing BCa (4). However, these methods are expensive and invasive, making it difficult for many patients to afford them, which may delay diagnosis (5). Therefore, as a noninvasive and inexpensive method, imaging techniques play an increasingly important role in the diagnosis of BCa (6). At present, magnetic resonance imaging (MRI), positron emission tomography (PET), and computed tomography (CT) are the conventional imaging methods for diagnosis before treatment (7). However, due to the complex and variable imaging features of BCa, it is difficult for radiologists to make an accurate BCa diagnosis based only on their experience. Therefore, there is an urgent need for better imaging methods to achieve a noninvasive and accurate diagnosis of BCa.

Deep learning (DL) is a rapidly developing field in machine learning (ML). Compared with classical ML algorithms, manual selection of features is not necessarily required in advance in DL. In contrast, the algorithm can learn the most relevant features for classification or prediction (8). In addition, it easily takes advantage of increases in the amount of available computation and data, with very little engineering by hand. This makes DL particularly useful for solving complex computational problems involving large-scale image classification, speech recognition, and many other domains (9, 10).

**Figure 1:**
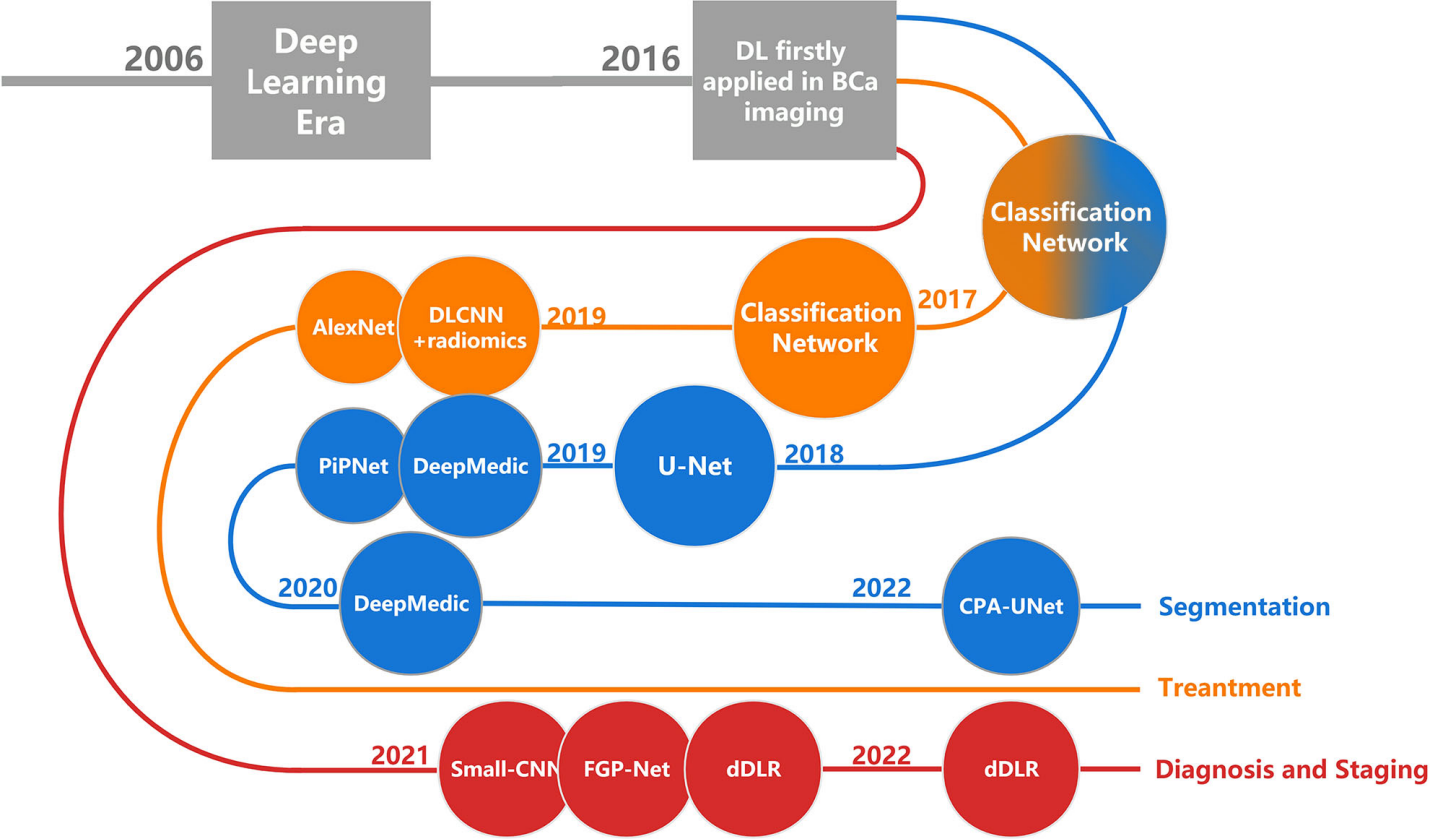
The development history of DL in BCa imaging. Each node corresponds to a research, named after the DL architecture that the research primarily used. DL, deep learning; BCa, bladder cancer.

Medical images contain a vast amount of data with extremely valuable signals and information, which is far beyond the ability of human beings to analyze. ML is naturally and rapidly used in this field because of its unique ability to integrate, analyze, and make predictions based on large amounts of data (11). As an emerging technology in recent years, DL has the potential to make better use of a large amount of data and provide better results (12, 13). In this review, we describe the research status of DL in the image segmentation, diagnosis, staging, and treatment response prediction of BCa (**Figure 1**). We are the first comprehensive review to present the current state of research on DL in BCa imaging. We focus on the purpose, DL methods, advantages, and limitations of the current research and discuss possible future directions in the field.

## Methods

**Figure 2:**
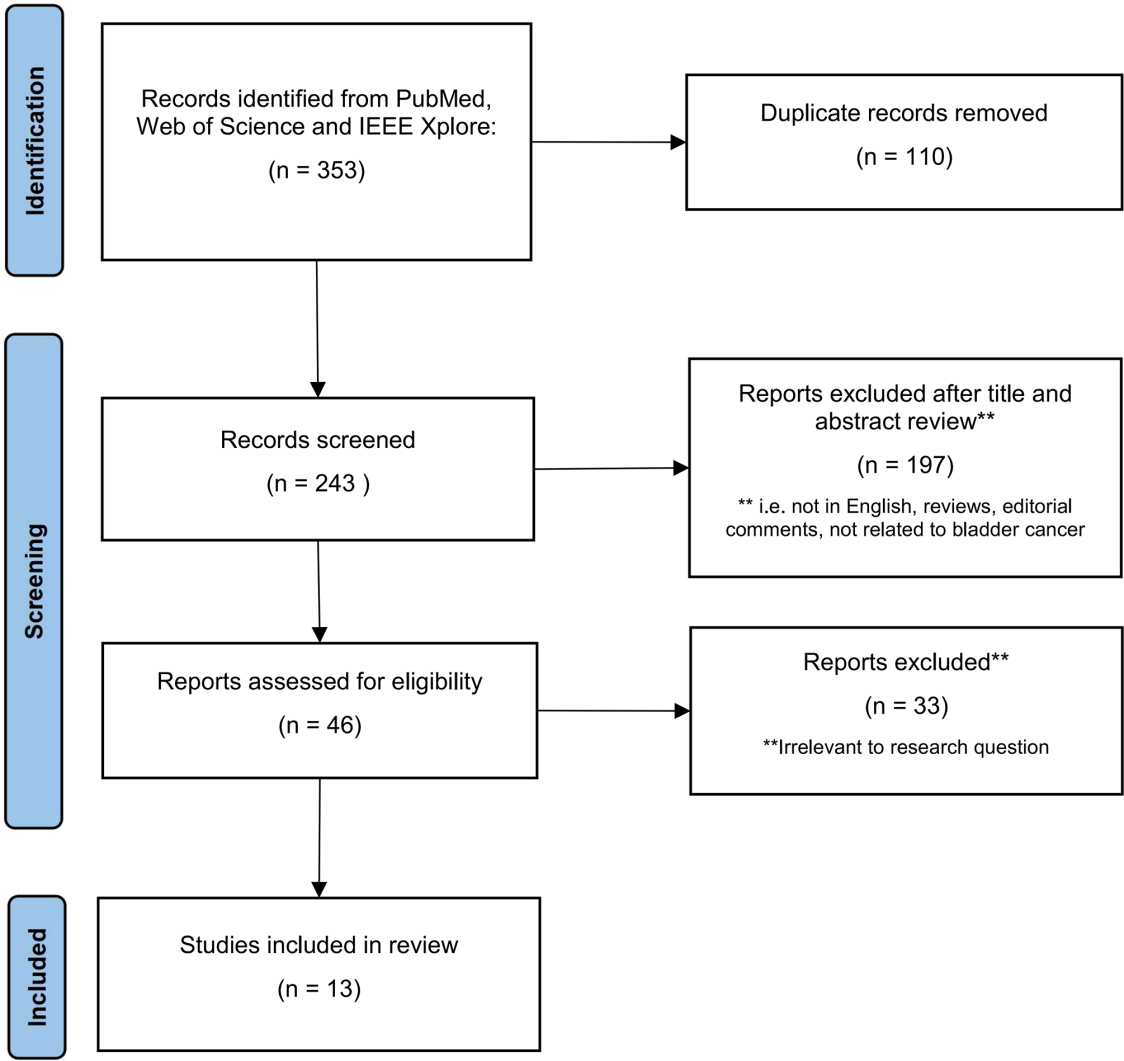
Summary of study selection process.

We conducted a literature search in the PubMed, Web of Science, and IEEE Xplore databases using the term “Bladder Cancer,” combined with the terms “Deep Learning”, “Diagnostic Imaging”, and “Medical Imaging”. In order to obtain articles that met the requirements of this review, we applied the following eligibility criteria: ① The paper is written in English; ② the paper is not a review article or editorial; ③ the paper is mainly related to BCa; ④ the paper discusses DL; and ⑤ the paper discusses imaging data. **Figure 2** illustrates the process of selecting articles based on the PRISMA criteria. To conduct our review, we extracted the names of the papers, authors, year of publication, DL modules, number of patients included, performance evaluation parameters, and many other features.

## Deep learning in bladder cancer segmentation

**Figure 3:**
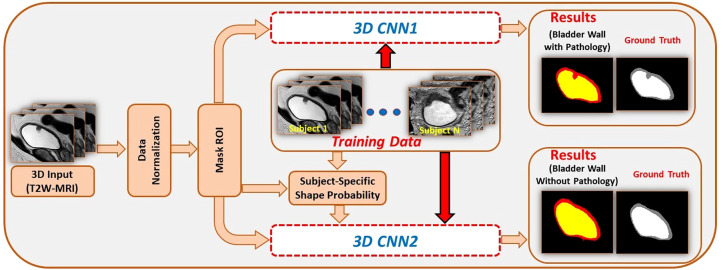
An example for bladder cancer image segmentation using deep learning. Image from Ref (16). Copyright © 2020, IEEE.

Medical image segmentation plays an important role in current medical imaging systems (14). In BCa, the accurate segmentation of normal bladder structures and tumor regions is an important step in tumor diagnosis and tumor stage evaluation (15). **Figure 3** illustrates the workflow of bladder cancer image segmentation using deep learning. The deep learning model is first trained by the training dataset and the ground truth label. Then the model can automatically analyze the input validation images and output the corresponding segmented images of all regions and compare them with ground truth for verification. However, as a hollow organ, the bladder undergoes various changes in position, shape, and volume. In addition, complex noise and artifacts are prevalent in medical images, which makes segmentation difficult (17–19). To date, many DL studies have focused only on the segmentation of the bladder wall (20–24). This is due to the high variability in tumor shape and intensity, making it difficult to distinguish between the bladder wall and a tumor. Therefore, it is more difficult to obtain accurate segmentation results than with normal bladder segmentation. In this review, we focus only on the literature that contains the segmentation of tumor regions (**Table 1**).

**Table 1:** Studies using deep learning approach for bladder cancer segmentation.

Author	Year	Modality	Number of patients (Train/Val/Test)	CNN structure	Target	Performance (validation or testing dataset)
Cha et al. (25)	2016	CT	62, LOOCV	A network contains 2 convolution layers, 2 locally connected layers, and 1 fully connected layer with level sets	Tumor	AVDIST = 4.7mm JACCARD = 36.3%
Dolz et al. (26)	2018	T2W MRI	40/5/15, LOOCV	U-Net with progressive dilated convolutional modules, 2D	IW/ OW/ Tumor	DSC (IW) = 0.9836 DSC (OW) = 0.8391 DSC (Tumor) = 0.6856 ASSD (IW) = 0.3517mm ASSD (OW) = 0.4299mm ASSD (Tumor) = 2.8352mm
Liu et al. (27)	2019	T2W MRI	40/-/7, n-fold CV	PiPNet (U-Net with progressive dilated convolutional modules and three prediction masks), 2D	OW/ Tumor	DSC (OW) = 0.8874 DSC (Tumor) = 0.9543
Hammouda et al. (28)	2019	T2W MRI	20, LOOCV	DeepMedic (a dual pathway CNN with a learnable adaptive shape prior model), 2D	IW/ OW/ Tumor	DSC (IW) = 0.9895 DSC (OW) = 0.9775 DSC (Tumor) = 0.9705 HD (IW) = 0.17mm HD (OW) = 0.18mm HD (Tumor) = 0.25mm
Hammouda et al. (16)	2020	T2W MRI	17, LOOCV	DeepMedic (two CNN network with a learnable adaptive shape prior model and CRF), 3D	IW/ OW/ Tumor	DSC (IW) = 0.9802 DSC (OW) = 0.9742 DSC (Tumor) = 0.9566 HD (IW) = 0.13mm HD (OW) = 0.19mm HD (Tumor) = 0.35mm
Yu et al. (29)	2022	T2W MRI	220/-/25,	CPA-Unet (a Unet for rough segmentation,a path augmentation structure for fine segmentation)	IW/ OW/ Tumor	DSC (IW) = 0.9819 DSC (OW) = 0.8224 DSC (Tumor) = 0.8740

AVDST, average distance; JACCARD, Jaccard similarity coefficient; DSC, Dice similarity coefficient; ASSD, average symmetric surface distance; HD, Hausdorff distance; IW, bladder inner wall; OW, bladder outer wall; LOOCV, leave-one-out cross-validation.

In 2016, Cha et al. (25) developed a network consisting of two convolution layers, two locally connected layers, and one fully connected layer, which is based on the well-known AlexNet (30) backbone. They then used level sets to perform minor refinements to the contour to identify the tumor boundary. However, these methods have many limitations, including a considerably slow process, sensitivity to initialization and image intensity, and independent pixel prediction. The achieved results were not significantly improved when compared with manual segmentation; therefore, they were quickly replaced by fully convolutional architectures.

U-Net (31) is undoubtedly one of the most successful methods in the fully convolutional architectures in image segmentation tasks, serving as the backbone of many new medical image segmentation methods. In 2018, Dolz et al. (26) added dilated convolutions to the U-Net model, where the dilation rate within each module progressively increased. The dilated convolutions can provide a larger receptive field that can leverage more contextual information. The increasing dilation rate allows the use of multi-scale information to better meet the segmentation requirements for both small and large objects. The model was trained and evaluated on T2-weighted (T2W) MR image datasets of 60 BCa patients and compared with the original U-Net, E-Net (32), and ERF-Net (33). The mean Dice similarity coefficient (DSC) values of their model were 0.98, 0.84, and 0.69 for the segmentation of the bladder inner wall, bladder outer wall, and tumor region, respectively, which were the best values of all the models trained. In addition, even though U-Net was improved with progressive dilated convolutional modules to avoid too much computation, the model’s inference time for the entire 3D volume is still less than 1 s. In 2019, Liu et al. (27) proposed a CNN architecture called the Pyramid in Pyramid Network (PiPNet), which is based on the U-Net model. The proposed PiPNet consists of a pyramid backbone similar to that of U-Net and adopts atrous spatial pyramid pooling (ASPP) of four parallel atrous convolutions with increasing dilation rates. In addition, the proposed model generates three prediction masks for the segmentation in the feature map of the last three layers to compute an overall loss function to extract multi-scale features. Depthwise separable convolution was used to improve the efficiency and performance of the model. The model was trained and evaluated on T2W MR images of 47 patients with BCa and compared with SegNet (34), U-Net, and Dolz’s (26) model. The DSC values were 0.89 and 0.95 for the outer wall and tumor, respectively, which were better than those of other models. Interestingly, in this study, Dolz et al.’s (26) model also achieved better results than the original, with DSCs of 0.86 and 0.92 for the outer wall and tumor, respectively. All models achieved better segmentation accuracy on tumors than on the bladder wall, contrary to the findings of Dolz et al. (26). Therefore, we believe that in the case of less data, different dataset quality and ground truth annotation methods have a greater impact on the performance of the trained model. Yu et al. (29) developed a Cascade Path Augmentation Unet (CPA-Unet) in 2022. They proposed a two-stage segmentation strategy and a hybrid loss function to improve the segmentation results. They first used U-Net for rough segmentation and then used the segmented image with the original image concatenated as a sample with two channels and input into the path augmentation structure (PA-Unet) for fine segmentation. The PA-Unet was based on the Path Aggregation Network (35), and the hybrid loss function incorporated the dice and cross-entropy losses, which can improve the performance (36). The CPA-Unet extracts multi-scale features more accurately, improves small target classification, and achieves better segmentation results than the U-Net, Prog Dilated (26), and PiPNet (27) networks.

These methods based on U-net improve the network performance through a more elaborate network design. However, these methods do not take advantage of the unique characteristics of BCa data and only improve the results by increasing network’s robustness. The advantages of these methods include better network characteristics and improved prediction results, which prove their effectiveness. However, as these methods are not specific in nature, which is not fundamentally different from other methods and networks in medical imaging, they do not make good use of data specificity when designing methods.

In addition to U-Net, another well-known CNN architecture for medical image segmentation, DeepMedic (37), has also been used for BCa segmentation. It can make better use of the geometric information of the bladder. Hammouda et al. (28) adopted a dual pathway 2D CNN to segment T2-weighted MRI images. In addition to inputting MRI image data, they also input subject-specific shape information that is adaptively built during segmentation. The adaptive shape prior (ASP) information comes from the results of co-aligning MRI images and ground truth images using an Affine transformation followed by a B-spline based transformation. The use of adaptive shape and contextual information significantly enhanced the segmentation performance, with DSC values of 0.99, 0.98 and 0.97 for the bladder inner wall, outer wall, and tumor, respectively. In 2020, Hammouda et al. (16) further improved their study. They extended their work to 3D bladder segmentation using T2W MRI. The proposed 3D CNN contains two branch networks. The first network aimed to segment the bladder wall with the tumor, and the second network only extracted the bladder. They used a 3D ASP model mixed with the original training data to feed the second network, and the outputs were refined using a fully connected conditional random field (CRF). The CRF can effectively reduce isolated small regions or small holes caused by local minima during training and noise in the input images. The performance of the proposed model significantly outperformed that of U-Net. These methods improved the results because the novelty of these methods changed from a simple network layer design to combining geometric information for segmentation.

When comparing the results of the existing segmentation works, we found that different literature often adopted different evaluation metrics. Most articles used the popular evaluation metric in medical image segmentation, the Dice coefficient (DSC). It can be computed as follows:


$$\text{DSC}=\frac{2\left|A\cap B\right|}{\left|A\right|+\left|B\right|}$$

DSC is a metric to assess the similarity between the predicted area and ground truth area based on the number of pixels of the overlapping region. A similar evaluation metric to it is the Jaccard index, which can be defined as:


$$\text{JACCARD}=\frac{A\cap B}{A\cup B}$$

However, region-based evaluation metrics are not sufficient to evaluate the segmentation of the bladder wall or to evaluate the contour consistency between the predicted area and ground truth area. Therefore, some articles included distance-based evaluation metrics, such as the average distance (AVDIST), the average symmetric surface distance (ASSD) and the Hausdorff distance (HD). AVDIST (25) is the average of the distances between the closest points of contours A and B and can be calculated as follows:


$${\text{AVDIST}}^{3D}\text{(A,B)}=\frac{1}{2}\left(\frac{\sum_{a\in A}\underset{b\in B}{\min}d(a,b)}{N_{A}}\right)+\left(\frac{\sum_{b\in B}\underset{a\in A}{\min}d(b,a)}{N_{B}}\right)$$

*N*_*A*_ and *N*_*B*_ denote the number of voxels on A and B, respectively. The function *d* is the Euclidean distance. The ASSD is also used to calculate the average distance between 2 contours, which can be defined as follows:


$$\text{ASSD(A, B)}=\frac{1}{\left|A\right|+\left|B\right|}\left(\sum_{a\in A}\underset{}{\underset{b\in B}{\min}d(a,b)+\sum_{b\in B}\underset{a\in A}{\min}d(b,a)}\right)\;$$

The HD is also a commonly used distance-based evaluation metric that is sensitive to segmentation boundaries. It can be computed using the following equation:


$$\text{HD(A, B)}=\max\left\{\overset{}{\underset{a\in A}{\max}}\left\{\overset{}{\underset{b\in B}{\text{min}}}\left\{d(a,b)\right\}\right\},\overset{}{\underset{b\in B}{\max}}\left\{\overset{}{\underset{a\in A}{\min}}\left\{d(a,b)\right\}\right\}\right\}$$

However, the use of diverse evaluation metrics makes it difficult to directly compare the performance of different models. In addition, metrics that are closely related to the clinical application such as model computation time should also be included. We believe that the adoption of consistent and comprehensive evaluation metrics, such as DSC and HD, can help us recognize the effects of different methods and make reasonable improvements.

In summary, these researches use different deep learning networks and algorithms to significantly improve the segmentation accuracy. Before deep learning methods were widely used, early literature used methods including Markov Random Fields, region growing, mathematical morphology, level-set, Chan-Vese model, geodesic active contour (GAC) and continuous max-flow algorithm for bladder segmentation (17–19, 38–47). And most of these researches were not able to segment tumor regions due to the limitations of algorithm and dataset size. In the only article that segmented the tumor region and used JACCARD as an evaluation criterion, they adopted a level-set-based method on a small dataset of ten patients, and the JACCARD of tumor regions extracted by it was 86.3% (45). The best DSC of tumor segmentation among the deep learning methods, on the other hand, reached 97.05% (28). For the segmentation of the bladder wall, the best DSC achieved by the method before deep learning was 87.28% (47). In contrast, the DSC of bladder wall segmentation of deep learning methods generally achieves over 90%. Deep learning methods have different innovations and produce satisfactory results that beyond traditional methods.

## Deep learning in bladder cancer diagnosis and staging

BCa is divided into non-muscle-invasive bladder cancer (NMIBC) and muscle-invasive bladder cancer (MIBC) according to whether the cancer invades the muscle (4). NMIBC accounts for approximately 75% of BCa cases and MIBC accounts for approximately 25%. MIBC is associated with a high degree of malignancy and a poor prognosis. The 5-year survival rate of MIBC patients after radical cystectomy is approximately 45-68%, whereas the survival time of MIBC patients with metastases generally does not exceed 2 years (48). Therefore, early and accurate diagnosis of BCa and assessment of the tumor stage are crucial for guiding clinical treatment and evaluating patient prognosis (49, 50).

In the past, the combination of artificial intelligence and radiomics has replaced traditional methods of manually defining the region of interest (ROI) and extracting image features and has achieved good results in the diagnosis and staging of BCa (51). However, DL can perform the above tasks automatically and achieve better results (**Table 2**). Yang et al. (52) proposed a small DL-CNN containing four convolutional and max-pooling layers to differentiate NMIBC from MIBC. The small DL-CNN was trained on their own database of 369 patients. In contrast, they developed eight well-known models that were pretrained on the ImageNet dataset. The results show that the possibility of overfitting for the small-CNN is minimized with a sensitivity of 0.722 and a specificity of 1.000. This may be because of the relatively low complexity of the model. Among the eight pretrained DL-CNNs, VGG16, VGG19, etc. (56) showed high performance, with an AUROC of 0.997-0.762. In general, DL-CNNs can achieve a favorable performance. However, in this study, an additional artificial enhancement step was required before the data were fed into the DL-CNN model rather than being fully automatic. This prevents the fully automated processing capability of DL from being fully exploited. Zhang et al. (53) used CT urography images of 441 patients from two medical centers to predict the muscular invasiveness of BCa. To date, this is a rare multicenter study of DL in BCa with a large dataset. The model is based on a novel 3D DL-CNN, a Filter-guided Pyramid Network (FGP-Net) (57). Dense blocks were applied to the network to enhance the transmission of features and alleviate vanishing-gradient problems, and discriminative filter learning (DFL) modules were used to enhance the mid-level representation by learning a bank of convolutional filters that capture class-specific discriminative patches. The network adopted a 2-channel input, and the input data consisted of a vertical superposition of the original and masked tumor regions. They compared the evaluation results of the model with those of two radiologists. Notably, they applied an external cohort evaluation to assess performance more rigorously (58). Although its final performance is not satisfactory and needs to be improved, the DL model can obtain slightly better, more objective, and more stable results compared with the results of the two radiologists. However, the objective results had another advantages. Radiologists may subjectively improve tumor staging in some ambiguous patients because of concerns about the negative consequences of losing MIBC, which may help in early clinical intervention. Liu et al. (54) adopted the ResNet18 (59) network for the diagnosis and staging of BCa based on MRI. They applied the super-resolution module and non-local attention module to improve the quality of MRI images and enhance the model’s ability to perceive features at longer distances.

**Table 2:** Studies using deep learning approach for bladder cancer diagnosis and staging.

Author	Year	Modality	Number of patients (Train/Val/Test)	CNN structure	Performance (validation or testing dataset)
Yang et al. (52)	2021	CT	369 patients,1200 images (70%/15%/15%)	A small convolutional network contains four conv_layer+max_pooling_layer stages/eight pretrained models, 2D	Accuracy (small) = 0.861 AUROC (small) = 0.998 Accuracy (VGG16) = 0.939 AUROC (VGG16) = 0.997
Zhang et al. (53)	2021	CT	183/110/73 (internal)/75 (external)	FGP-Net (a novel convolutional network contains Dense Blocks and DFL modules), 3D	AUC (internal) = 0.861 Accuracy (internal) = 0.795 AUC (external) = 0.791 Accuracy (external) = 0.747
Liu et al. (54)	2022	T2W MRI	51/8/16	ResNet18 with the super-resolution module and the Non-local attention module, 2D	Sensitivity = 94.74
Taguchi et al. (55)	2021	T2W MRI	68	The denoising Deep Learning Reconstruction (dDLR)	–

AUC, area under curve; Sensitivity=TP/(TP+ FN).

In addition to diagnosis, DL can be used to improve other parts of the imaging workflow, such as removing image noise and indirectly improving diagnostic capabilities in conjunction with other systems. The vesical imaging reporting and data system (VI-RADS) (60) is a tool for evaluating BCa staging using MRI images. Taguchi et al. (55) used a convolutional neural network to improve the signal-to-noise ratio in high-spatial-resolution images. Although they did not develop the network themselves, this study also showed the potential of DL in assisting in BCa diagnosis.

## Deep learning in bladder cancer treatment assessment

Neoadjuvant chemotherapy has been shown to improve overall survival for patients with BCa (61). However, not all patients benefit from neoadjuvant treatment and instead suffer from severe side effects (62). Therefore, it is important to assess changes in tumor size and treatment response early to help doctors make personalized treatment plans. Nevertheless, there are two major problems with the current clinical treatment assessment. First, although accurate, surgery may not be appropriate for patients undergoing chemotherapy. Second, the current World Health Organization (WHO) criteria (63) and Response Evaluation Criteria in Solid Tumors (RECIST) (64) are inaccurate. Neither set of criteria address three-dimensional (3D) measurements, and the results are heavily influenced by observer experience, especially for tumors with complex and irregular shapes (65). At the same time, because organs and tumors are not rigid bodies, they will have different deformations in the human body, making the design of direct networks for ML very difficult. These problems make ordinary ML methods not particularly adaptable, and therefore drive the progress of DL methods in this field. DL has been recognized as a powerful tool to solve these problems (**Table 3**).

**Table 3:** Studies using deep learning approach for bladder cancer treatment.

Author	Year	Modality	Number of patients (Train/Val/Test)	CNN structure	Performance (validation or testing dataset)
Cha et al. (25)	2016	CT	62, LOOCV	A network contains 2 convolution layers, 2 locally connected layers, and 1 fully connected layer.	AUC = 0.73
Cha et al. (66)	2017	CT	82	A network contains 2 convolution layers, 2 locally connected layers, and 1 fully connected layer. Each layer contains 16 kernals.	AUC = 0.73
Wu et al. (67)	2019	CT	73/9/41	The basic network contains 2 convolution layers, 2 locally connected layers, and 1 fully connected layer.	AUC (basic-random weights) = 0.73 AUC (basic-pretrained weights) = 0.79 AUC (DL-CNN-1) = 0.72 AUC (DL-CNN-2) = 0.86 AUC (DL-CNN-3) = 0.69 AUC (C1 Frozen) = 0.81 AUC (C1,C2 Frozen) = 0.78 AUC (C1,C2,L3 Frozen) = 0.71
Cha et al. (68)	2019	CT	123, LOOCV	DL-CNN with a radiomics assessment model	AUC (CDSS-T only) = 0.80 AUC (with CDSS-T) = 0.77 AUC (No CDSS-T) = 0.74

AUC, area under the curve; LOOCV, leave-one-out cross-validation.

Cha et al. (25) used the network they developed to segment and measure the gross tumor volume (GTV) from CT images to predict treatment response. As described in the bladder segmentation section, classification-based networks cannot accurately segment tumors because of their limitations, particularly those that shrink after treatment. Their DL-CNN was comparable to radiologists’ manual predictions. In 2017, Cha et al. (66) developed a DL-CNN with a structure similar to that in previous studies. However, DL-CNN was used to predict the response to neoadjuvant chemotherapy in this study. They first used their auto-initialized cascaded level set (AI-CALS) (69) system to segment the tumor region. They then paired ROIs extracted from pre- and post-treatment tumor regions of the same patient’s scans to form 6700 image pairs. They compared the model with two radiomic feature-based approaches. Owing to their relatively simple DL-CNN structure, the three methods they tested achieved similar results and were also similar to the manual methods. However, it also demonstrates the potential of DL techniques in predicting the treatment response. In 2019, Wu et al. (67) developed seven DL-CNNs based on a previous study (66) and adopted the same image-processing method (66). They modified the filter size, filter stride, and padding type of convolutions and max pooling performed in layers C1 and C2 to develop three different models, and developed two models by freezing different layers. Furthermore, they pretrained the model on the CIFAR10 (70) image set. Only one network variation (DL-CNN-2, C1 convolution filter stride 1→2, C2 max pooling size 3×3→2×2, stride 2→1) exhibited significant performance improvements. The performance of the DL-CNN generally decreased as more layers were frozen, but there was a slight improvement in performance when the C1 layers were frozen. This may be because the subsequent layers are designed to capture more specific features, such as bladder lesions. The pretrained network achieved better performance, but it was better to pretrain with data related to the training images. Overall, they demonstrated that the use of DL-CNN can match or even exceed the level of doctors, and using deeper DL-CNN models and making more effective adjustments to network structures can further improve its performance in the future. Recently, Cha et al. (68) developed a computerized CT-based decision-support system for MIBC treatment response assessment (CDSS-T) based on their previous work (56). They followed the segmentation system and their previously developed DL-CNN combined with a radiomics assessment model. A combined score from the DL-CNN and radiomic model was used to assist physicians in the assessment of the treatment response. With the help of the CDSS-T, 12 physicians improved the assessment accuracy for evaluating the neoadjuvant chemotherapy response in MIBC. This is the first observer study to use a CAD system for this purpose. Interestingly, the accuracy rate of the CDSS-T alone was higher than that of using CDSS-T to assist physicians in assessment. This shows that doctors’ experience and trust in using the system still needs to be cultivated, which is also one of the key issues to be overcome in the future clinical application of DL.

## Challenges and future directions

DL is a state-of-the-art technology and popular research area in medical imaging. Its performance is comparable to that of human experts in many studies and applications and it has good development prospects and potential (71). However, research on DL in BCa is still in its infancy, and there are still many shortcomings compared to other fields with mature applications.

### For data

The imaging diagnosis of BCa by clinicians often requires the integration of various imaging data, such as CT and different sequences of MRI images. Although CT is the most commonly used imaging technique for the diagnosis of BCa, MRI has been shown to be more effective, especially in staging, because of the increased soft-tissue contrast resolution. Diffusion-weighted imaging (DWI) and dynamic contrast enhancement (DCE) are far more useful for assessing tumor invasiveness and infiltration into surrounding structures. However, most of the current DL studies on BCa imaging still use CT as the original data. Moreover, all studies using MRI have chosen T2WI sequences, and there is a lack of studies on DWI and DCE sequences. Combining DL with the most appropriate as well as the most advanced techniques in BCa imaging will be a research direction. In addition, based on CT or MRI, most data currently used in BCa studies focus on only one modality of medical imaging. In recent years, many studies have shown that processing multiple modalities simultaneously can significantly improve the performance of DL models (26, 72, 73).

We can also attempt to improve performance by combining imaging-based assessment with other available clinical data, such as genomics and pathology. Multimodal approaches have been shown to outperform unimodal ones (74). In fact, in both natural and medical image processing, multimodal fusion is becoming a mainstream and effective trend. BCa are heterogeneous at the molecular level, and different molecular classifications may be useful to stratify patients for prognosis or response to treatment. The inclusion of multimodal information helps to complement the shortcomings of BCa imaging in these areas. However, due to various reasons, such as the small number of BCa open datasets, there are not many multi-modality processing methods used in the research of DL in BCa. In addition, the limited quantity of medical image data restricts the development of DL. The amount of data significantly affects the performance of DL models. Transfer learning (75) and data augmentation can improve performance to some extent, but they cannot replace the need for a large dataset. To date, the datasets of many studies of DL in BCa have been so small that they do not even have independent validation or test sets, which biases the assessment of the model performance. In addition, the different scanning methods and equipment adopted by different hospitals make the established models difficult to use across institutions, which also limits the clinical application of DL. In this case, it is necessary to use semi-supervised or self-supervised methods to process data. However, the application of these methods for BCa is limited, highlighting the need for future research. In this case, we expect increasing data diversity, multimodal methods, and more comprehensive BCa datasets including multi-center data or a nationwide BCa imaging database to significantly advance the field.

### For algorithm

Most of the DL models used in the current research only stay in the application of existing networks and lack optimization of the imaging characteristics of BCa. The BCa data have many unique structures, including their unique geometry, empty structure, and other characteristics. However, in the current research field on BCa, these characteristics are not well utilized. Compared with other ML methods, DL is a complex black box. To optimize this model in the future, it is important to reflect doctors’ ideas and experiences in the diagnosis and treatment of diseases in the DL model and improve its interpretability. Only when the doctor can understand the reason why the DL model makes the assessment can the model better assist the doctor in decision-making. Furthermore, many state-of-the-art results in the field of DL, such as self-supervised learning, pre-training models, transformers, and contrastive learning, have not yet been applied in the field of BCa research, which could be the subject of our future research.

### For application

There are many application scenarios and research directions of DL that people can explore in BCa. For example, there are various pathological types of BCa, including urothelial carcinoma and squamous cell carcinoma. NMIBC and MIBC can also be divided into many molecular subtypes according to the MD Anderson Cancer Center (MDA) (76), Cancer Genome Atlas (TCGA) (77), and other classification criteria. Based on the above criteria, a more complex classification of BCa can be attempted using medical imaging. In addition, DL can be used to predict patient prognosis through medical imaging. Whether DL can predict the outcome of surgical treatment for BCa or be applied to ROI extraction, feature extraction, and feature modelling in radiomics remains unclear. At present, a large amount of research is still focused on image segmentation, and we believe that the development of DL can help doctors in more ways.

## Conclusions

This study reviews the applications of DL in BCa imaging. As a potential technology, DL has extremely broad application prospects in BCa. Limited by the small number of studies in this field, we provide a detailed review of the existing studies, but lack more evidence to demonstrate more possibilities of DL in BCa imaging. However, in the era of increasing emphasis on precision medicine and individualized diagnosis and treatment, how to give full play to the advantages of DL and transform it into a means that can effectively help physicians in clinical diagnosis and treatment will be the direction of our future research. The powerful potential demonstrated by DL is expected to bring about a new revolution in BCa management.

## Author contributions

ML conceived and reviewed the manuscript. ZJ helped with the revising and provided insightful suggestions on the manuscript. All authors have contributed to the manuscript and approved the submitted version.

## Funding

This work was supported by a project of the National Facility for Translational Medicine (Shanghai) (TMSK-2021-118).

## Conflict of interest

The authors declare that the research was conducted in the absence of any commercial or financial relationships that could be construed as a potential conflict of interest.

## Publisher’s note

All claims expressed in this article are solely those of the authors and do not necessarily represent those of their affiliated organizations, or those of the publisher, the editors and the reviewers. Any product that may be evaluated in this article, or claim that may be made by its manufacturer, is not guaranteed or endorsed by the publisher.
